# A174 THE DIRECT HEALTHCARE COST OF INFLAMMATORY BOWEL DISEASE IN CANADA: A POPULATION-BASED ANALYSIS OF ADMINISTRATIVE DATA

**DOI:** 10.1093/jcag/gwae059.174

**Published:** 2025-02-10

**Authors:** S Coward, E I Benchimol, C N Bernstein, A Avina-Zubieta, A Bitton, F Hoentjen, E Kuenzig, N Lu, J Leal, C Ma, S Murthy, K Novak, Z Nugent, A Otley, R Panaccione, J Peña-Sánchez, H Singh, L Targownik, G G Kaplan

**Affiliations:** University of Calgary, Calgary, AB, Canada; The Hospital for Sick Children, Toronto, ON, Canada; University of Manitoba Max Rady College of Medicine, Winnipeg, MB, Canada; The University of British Columbia, Vancouver, BC, Canada; McGill University, Montreal, QC, Canada; University of Alberta, Edmonton, AB, Canada; The Hospital for Sick Children, Toronto, ON, Canada; Arthritis Research Canada, Richmond, BC, Canada; University of Calgary, Calgary, AB, Canada; University of Calgary, Calgary, AB, Canada; University of Ottawa, Ottawa, ON, Canada; University of Calgary, Calgary, AB, Canada; University of Manitoba, Winnipeg, MB, Canada; Dalhousie University, Halifax, NS, Canada; University of Calgary, Calgary, AB, Canada; Department of Community Health and Epidemiology, University of Saskatchewan, Saskatoon, SK, Canada; University of Manitoba, Winnipeg, MB, Canada; University of Toronto, Toronto, ON, Canada; University of Calgary, Calgary, AB, Canada

## Abstract

**Background:**

The prevalence of inflammatory bowel disease (IBD) in Canada is rising rapidly, with an estimated 0.86% of the population living with IBD in 2025. With this rise in prevalence comes a rise in the direct costs to healthcare systems.

**Aims:**

To assess direct healthcare costs associated with IBD in Canada.

**Methods:**

We analyzed population-based administrative healthcare costing data from AB, BC, MB, and SK from fiscal year (FY) 2010/11 to 2016/17. Costs were adjusted to 2020 CAD$ using the Consumer Price Index. Average annual costs were calculated for: annual per IBD person cost (All), and medication costs (on a biologic ± other IBD-related medications, and only other IBD-related medication [eg. mesalamine] (AB,BC,MB)). Per event costs were calculated by outcome: IBD-related hospitalization or surgery (AB,MB), emergency department visit (AB, BC), and colonoscopy (AB,BC). We calculated the average annual percentage change (AAPC) with 95% confidence intervals (CI) using weighted costs in log-gamma models. Autoregressive moving average models to forecasted costs to FY2025/26 with 95% prediction intervals (PI).

**Results:**

In FY 2016/17, the annual average cost per IBD person was $11186 (95%CI:11052,11320), significantly increased from FY2010/11 (5.12%;95%CI:4.75,5.48). Biologics were the largest cost, accounting for 45.11% (95%CI:44.23,45.98) of the total costs and significantly increasing (2.13%; 95%CI:1.43,2.84). Costs for other IBD medications significantly decreased (−2.00%; 95%CI:−3.47,−0.51), contributing 5.46% (95%CI:5.09,5.64) of total costs. Costs of emergency department visits and colonoscopies significantly increased, while costs for IBD-related hospitalizations and surgeries remained stable. By FY2025/26, the annual cost per IBD person is forecasted to be $15345 (95%PI:14915,15775).

**Conclusions:**

The direct healthcare costs of IBD are rising, largely driven by the costs of biologics. As IBD prevalence continues to grow, the burden on healthcare systems is expected to escalate. Proactive measures are essential to address this burden and ensure individuals with IBD receive the necessary care.

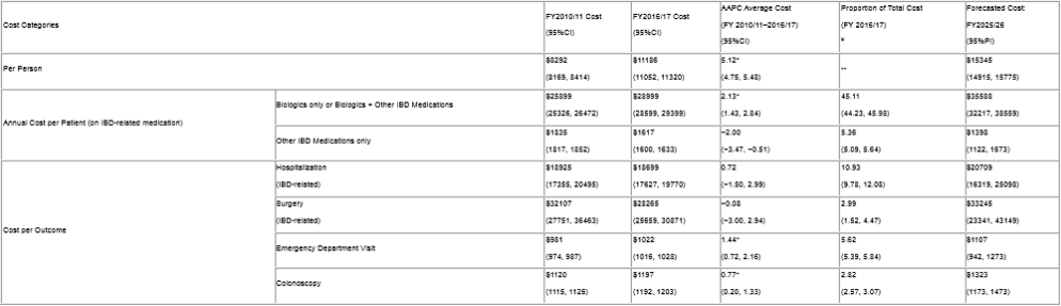

¥Proportions will not add up to 100% as individuals can be captured in multiple cost categories each year (e.g., an IBD-related surgery cost is also a hospitalization). * p<0.05

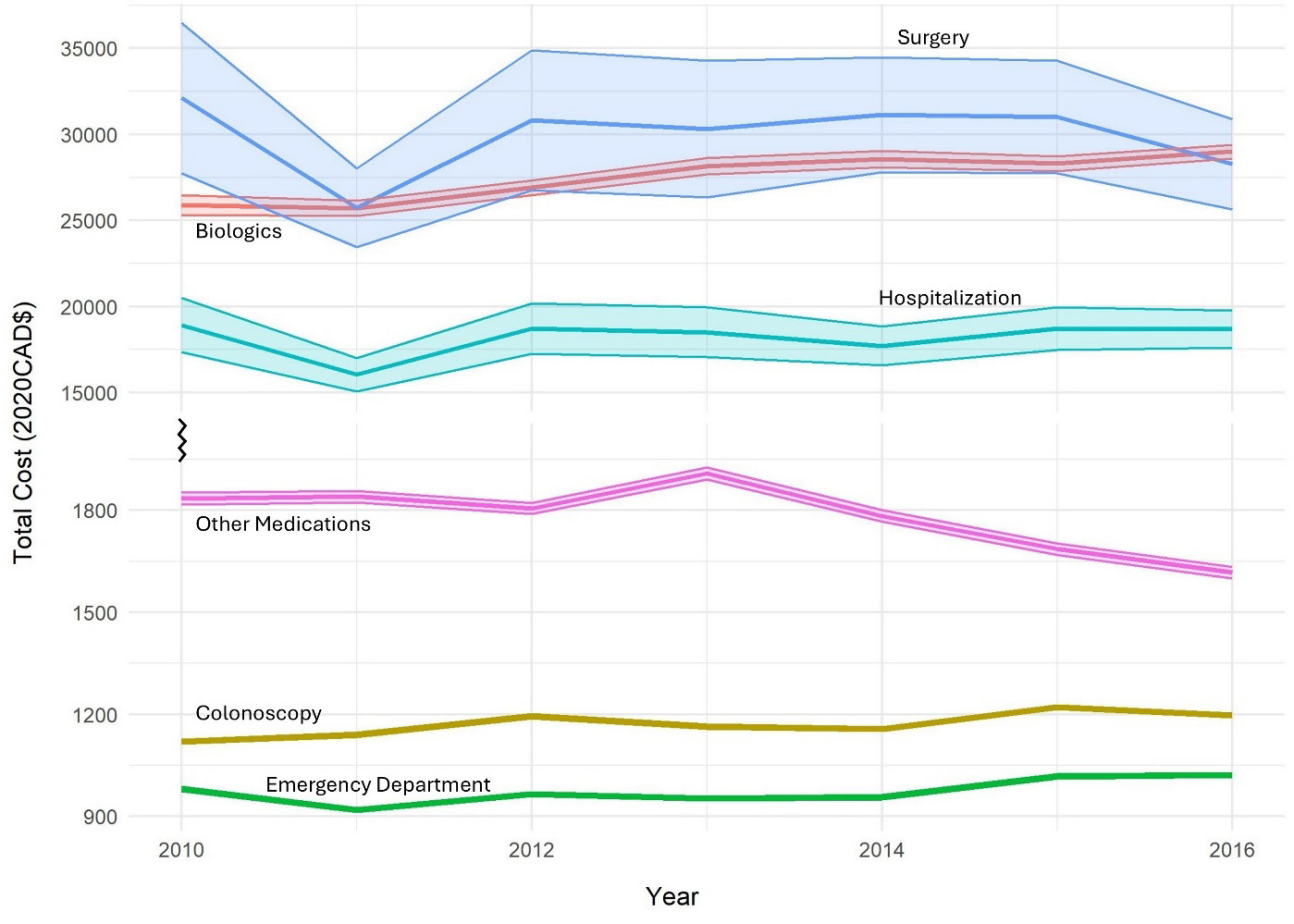

**Funding Agencies:**

CCC, CIHR

